# Spatial variability of saturated hydraulic conductivity and its links with other soil properties at the regional scale

**DOI:** 10.1038/s41598-021-86862-3

**Published:** 2021-04-15

**Authors:** Boguslaw Usowicz, Jerzy Lipiec

**Affiliations:** Institute of Agrophysics, Polish Academy of Sciences, Doswiadczalna 4, 20-290 Lublin, Poland

**Keywords:** Environmental sciences, Hydrology

## Abstract

Saturated hydraulic conductivity (K) is a key property for evaluating soil water movement and quality. Most studies on spatial variability of K have been performed soil at a field or smaller scale. Therefore, the aim of this work was to assess (quantify) the spatial distribution of K at the larger regional scale in south-eastern Poland and its relationship with other soil properties, including intrinsic sand, silt, and clay contents, relatively stable organic carbon, cation exchange capacity (CEC) and temporally variable water content (WC), total porosity (FI), and dry bulk density (BD) in the surface layer (0–20 cm). The spatial relationships were assessed using a semivariogram and a cross-semivariogram. The studied region (140 km^2^) with predominantly permeable sandy soils with low fertility and productivity is located in the south-eastern part of Poland (Podlasie region). The mean sand and organic carbon contents are 74 and 0.86 and their ranges (in %) are 45–95 and 0.002–3.75, respectively. The number of individual samples varied from 216 to 228 (for K, WC, BD, FI) to 691 for the other soil properties. The best fitting models were adjusted to the empirical semivariogram (exponential) and the cross-semivariogram (exponential, Gaussian, or linear) used to draw maps with kriging. The results showed that, among the soil properties studied, K was most variable (coefficient of variation 77.3%) and significantly (*p* < 0.05) positively correlated with total porosity (r = 0.300) and negatively correlated with soil bulk density (r = – 0.283). The normal or close to the normal distribution was obtained by natural logarithmic and root square transformations. The mean K was 2.597 m day^−1^ and ranged from 0.01 up to 11.54 m day^−1^. The spatial autocorrelation (range) of K in the single (direct) semivariograms was 0.081° (8.1 km), while it favourably increased up to 0.149°–0.81° (14.9–81 km) in the cross-semivariograms using the OC contents, textural fractions, and CEC as auxiliary variables. The generated spatial maps allowed outlining two sub-areas with predominantly high K above 3.0 m day^−1^ in the northern sandier (sand content > 74%) and less silty (silt content < 22%) part and, with lower K in the southern part of the study region. Generally, the spatial distribution of the K values in the study region depended on the share of individual intrinsic textural fractions. On the other hand, the ranges of the spatial relationship between K and the intrinsic and relatively stable soil properties were much larger (from ~ 15 to 81 km) than between K and the temporally variable soil properties (0.3–0.9 km). This knowledge is supportive for making decisions related to land management aimed at alteration of hydraulic conductivity to improve soil water resources and crop productivity and reduce chemical leaching.

## Introduction

Saturated hydraulic conductivity (K) is a key characteristic of soil, describing the rate of water flow, pathways of water movement partitioning precipitation and irrigation water into surface runoff and retention in the soil^1,2^, and the soil water dynamics in the soil profile^3^^.^ High K leads to rapid water infiltration and drainage^4,5^ and reduced time for allowing agrochemicals to be retained in the soil matrix^6^, whereas low K increases surface runoff and erosion^7,8^. Thereby, K helps farmers to apply an appropriate amount of irrigation water^9^. Furthermore, it affects air-filled porosity^10,11^ influencing nutrient transformations and uptake by plants^12,13^. Due to the numerous contributions, K is often used as a measure of soil physical quality (e.g.^12^). Also, it is a key parameter in mathematical models for predicting soil hydraulic behaviour^2,14,15^.

The K value depends largely on the pore size distribution (PSD), especially on the share and continuity of relatively large pores (macropores)^9,16–20^. In a study conducted by Kim et al.^21^, the area of the largest pores explained almost 80% of variability in soil saturated hydraulic conductivity. As shown by Centeno et al.^22^, macro-porosity can be used as a proxy to estimate the spatial variation of K.

Owing to the high sensitivity to pore size distribution influenced by soil texture and management practices, K displays relatively high spatial variability^9,15,20,22,23^. Therefore, knowledge of the spatial distribution of K is essential in selection of the most appropriate localised management practices and amendments to improve water use efficiency in agriculture and to minimise the use and leaching of chemicals^24–27^. The spatial distribution of soil properties, including saturated hydraulic conductivity, can be assessed by classical and spatial statistics. The classical statistics can adequately analyse variables that are independent of space^28^. However, when the random variation occurs, geostatistical analysis including direct semivariograms and cross-semivariograms is appropriate^29,30^. Semivariograms define the dependence of the difference between values of a given variable on the distance between sampling locations and, hence, the spatial structure of the variation. They aid in designing a sampling setup with an amount of samples required for satisfactory regionalization of soil properties^31,32^. Once various variables are linked, their combined spatial designs can be evaluated by cross-semivariograms. Semi-variogram and cross-semivariogram data and maps obtained using krging and cokriging techniques. Cokriging also allows distinguishing time-consuming and/or expensive variables from those that are more easily measured or available in soil databases. When K shows spatial random variation, the use of both classical statistics and geostatistical models are recommended^30^.

Numerous studies on the spatial variability of soil K have been performed to date at a short scale (< 25 m) (e.g.^33,34^) or a field scale^35^. However, the variability at a larger regional scale is poorly understood, as suitable spatial characterisation of highly heterogeneous K requires a large number of laborious, time-consuming, and expensive direct measurements^9,36,37^. Therefore, the objective of this study was to determine the spatial distribution of K at the regional scale using a semivariogram and a cross-semivariogram and its relationship with soil properties that affect conductivity and can be more easily measured or obtained from existing soil databases.

## Materials and methods

### Study area and sampling

Study region with an area of about 140 km^2^ is situated in a flat area within Łuków Plain, south-eastern Poland. The height differences in the shallow and often wet river (Krzna) valleys do not exceed 10 m. The study region has mostly low productive Podzol soils^38^ derived from sandy and sandy loams of glacial origin. About 80% of the area in the region is used in agriculture, with 62.3% and 18.2% of arable lands and grasslands, respectively. Forests cover only 13.5% of the study area, mostly in the south-eastern and western parts. The climate is largely influenced by the western circulation and polar sea air (about 65% days a year). The average annual air temperature in the region is about 7.3 °C. July and January are the warmest and coldest months with respective mean temperatures of 17.7 and − 3.6 °C. The annual amplitude (the difference between max and min) of air temperature is 21.3 °C and 23.4 °C when calculated from the differences of the average temperature of the hottest (July) and coldest (January) months in individual years. The greatest amount of precipitation is recorded in June and July (more than 70 mm) and the lowest values (less than 30 mm) are noted in January, February, and March. This indicates significant predominance of summer rainfall (212 mm) over winter rainfall (83 mm). The sum of rainfall in the growing season (April–September), i.e. 350.9 mm, constitutes 65.4% of the annual total. During the 50 years under consideration, it ranged from 224 to 530 mm.

### Tested soil properties

**Figure 1:**
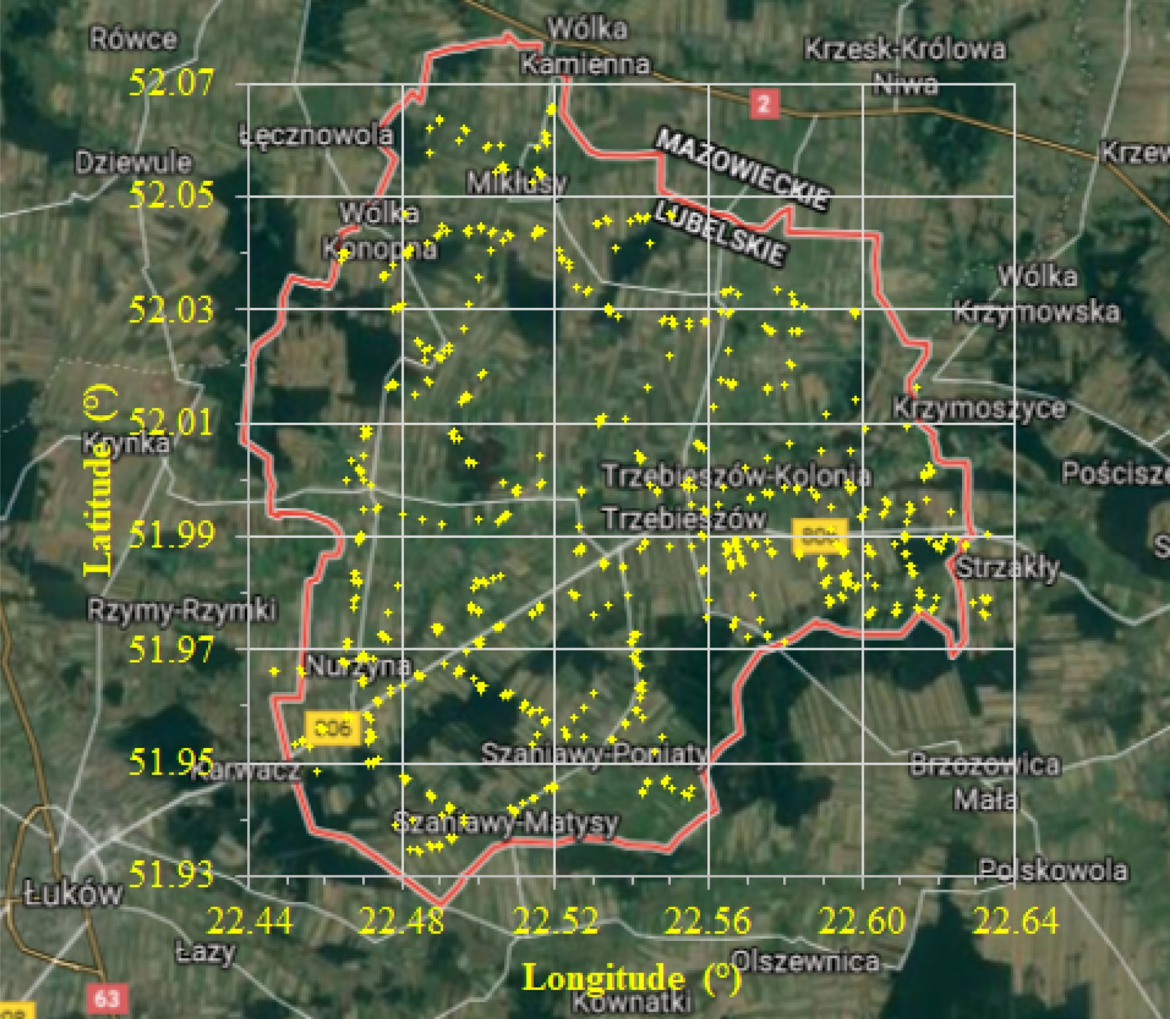
Location of sampling points in the study region. Background maps from “Map data: Google, TerraMetrics,Dane mapy ©2021 Google Polska. (https://www.google.pl/maps/place/Trzebiesz%C3%B3w/@51.9976784,22.3932616,28111m/data=!3m2!1e3!4b1!4m5!3m4!1s0x4721fbd1e8dac53f:0x77c729fd11bcd3f3!8m2!3d52.0122717!4d22.5207958), accessed 31 March 2020. The background maps were modified using Microsoft Office PowerPoint 2019.

Soil samples were randomly collected from the 0–20 cm layer into cloth bags and 100 cm^3^ (5.02 cm diameter and 5.05 cm high) steel cylinders immediately after harvesting cereals (August). Figure 1 shows the spatial distribution of the soil sampling points. The number of individual samples varied from 216 to 228 (for K, WC, BD, FI) to 691 for the other soil properties. At each randomly selected point we collected from a few to a dozen samples around, which we used in the geostatistical analysis.

Soil dry bulk density (Mg m^−3^) was determined with the gravimetric method from the ratio of the mass of soil dried at 105 °C to the soil volume of 100 cm^3^^39^. The gravimetric water content (WC_grav_) was determined from the ratio between water mass and mass of the soil after drying using the same cylinders as for determination of dry bulk density. Soil water content was also measured using a TDR meter (WC_TDR_) close to the sampling sites. Saturated hydraulic conductivity was measured with the constant head method in soil samples with a volume of 100 cm^3^ in a laboratory permeameter (Eijkelkamp Agrisearch Equipments, The Netherlands)^40^. The method assumes that K does not depend on the hydraulic head difference and the soil is the only resistance to water flow. Particle size distribution was analysed using the sieving and hydrometer method^41^. Soil organic matter was determined based on wet oxidation with K_2_Cr_2_O_7_ according to Tiurin's procedure^41^. The soil pH (in H_2_O) was determined potentiometrically using a composite electrode. Particle density (Mg m^−3^) was determined with the pycnometric method^42^. The total porosity (m^3^ m^−3^) was calculated as a ratio of 1 − bulk density/particle density^43^.

### Data analysis

#### Classical statistics

Basic statistics with the mean, standard deviation, coefficient of variation, minimum, maximum, skewness, and kurtosis were calculated for each soil property. Both kurtosis and skewness values of 0 indicate in general symmetrical distribution with a similar right tail (positive) and left tail (negative) of the distribution curve. When one tail is longer than the other, the distribution is asymmetric. As shown by Dahiya et al.^44^, the variability of the soil properties was categorised as low (0–15%), medium (15–75%), and high (> 75%). Pearson correlation coefficients between the soil variables were determined. The results were analysed using STATISTICA 12 PL (StatSoft 2019) and GS + 10^45^. Data normality was assessed using the Cumulative Frequency Distribution^45^. If the distribution was not normal the natural logarithmic and root square transformations were used to ensure the normal or close to the normal distribution.

#### Geostatistical methods

##### Semivariograms and cross-semivariograms

It is assumed that values of soil properties or physical quantities measured in a given point are dependent (similar, correlated) on other points. In this approach, similarity is described by half of the value expected from the difference between the value of *Z*(*x*) of the variable at point *x* and the value of *Z*(*x* + *h*) at a point distant by vector *h*. A variable whose values correspond to values *Z*(*x*) is "regionalised". This variable has a random aspect, which takes into account local anomalies, including a structural aspect reflecting the multiscale trend of the phenomenon (trend). The analysis of this variable consists of identifying the structure of the variation. Three phases of the analysis can be distinguished: preliminary examination of collected data and evaluation of basic statistics, calculation of the empirical variant of the considered regionalised variable, and adjustment of the mathematical model to the course of the empirical variant. The knowledge of the first two statistical moments of random functions is required: the first (expected value) and the second (variance) moment. It is also required that the examined process is stationary, i.e. it does not change its properties when changing the beginning of the time scale. In the case of fulfilment of the stationary process, the random function *Z*(*x*) is defined as the second order stationary and then the expected value exists and does not depend on the position, and the experimental semivariogram *γ*(*h*) (for a single variable *z*_1_) or the cross-semivariogram $\gamma_{12} \left( h \right)$ (for two variables *z*_1_ and *z*_2_) for distance *h* are calculated from the following equations^45^:

$$\begin{aligned} \gamma \left( h \right) & = \frac{1}{2N\left( h \right)}\mathop \sum \limits_{i = 1}^{N\left( h \right)} [z_{1} \left( {x_{i} } \right) - z_{1} \left( {x_{i} + h} \right)]^{2} \\ \gamma_{12} \left( h \right) & = \frac{1}{2N\left( h \right)}\mathop \sum \limits_{i = 1}^{N\left( h \right)} \left[ {z_{1} \left( {x_{i} } \right) - z_{1} \left( {x_{i} + h} \right)} \right] \cdot \left[ {z_{2} \left( {x_{i} } \right) - z_{2} \left( {x_{i} + h} \right)} \right] \\ \end{aligned}$$

where *N*(*h*) is the number of pairs of points with values of [*z*_1_(*x*_*i*_), *z*_1_(*x*_*i*_ + *h*)], [*z*_2_(*x*_*i*_), *z*_2_(*x*_*i*_ + *h*)], distant by *h*.

Three characteristic parameters for the semivariograms and cross-semivariograms are distinguished: nugget effect, sill, and range. When the value of the semivariograms increases not from zero but from a certain value, this value is called the nugget effect. It expresses the variability of the examined physical quantity at a scale smaller than the sampling interval and/or accuracy of measurement. A value at which no further increase in the semivariograms is observed (approximately equal to the sample variance) is called sill, while the distance from zero to the point where the semi- or cross-semivariogram reaches 95% of the sill value is called a range. The latter expresses the greatest distance at which the sampled values are auto- or cross-correlated.

For semi- and cross-semivariograms determined empirically, the following mathematical models were fitted using the least squares method^45^:

- The linear isotropic model describes a straight line variogram. There is no sill in this model; the range *A*_0_ is defined arbitrarily to be the distance interval for the last lag class in the variogram. The formula used is:
  $$\gamma \left( h \right) = C_{0} + \left[ {h\left( {{\raise0.7ex\hbox{$C$} \!\mathord{\left/ {\vphantom {C {A_{0} }}}\right.\kern-\nulldelimiterspace} \!\lower0.7ex\hbox{${A_{0} }$}}} \right)} \right]$$
- The exponential isotropic model. The formula used for this model is:
  $$\gamma \left( h \right) = C_{0} + C \cdot \left[ {1 - e^{{ - \frac{\left| h \right|}{{A_{0} }}}} } \right]\,\,\,\,\,\,\,\,\,\,\,\,\,\,\,\,\,\left| h \right| > 0$$
- The Gaussian isotropic model. The formula used for this model is:
  $$\gamma \left( h \right) = C_{0} + C \cdot \left[ {1 - e^{{ - \frac{\left| h \right|}{{A_{{{}^{0}}}^{2} }}^{2} }} } \right]\,\,\,\,\,\,\,\,\,\,\,\,\,\,\,\,\,\left| h \right| > 0$$
  where: *γ*(*h*) semivariance for internal distance class *h*, *h*—lag interval, *C*_0_—nugget variance ≥ 0, *C*—structural variance ≥ *C*_0_, *A*_0_—range parameter. In the case of the linear model, there is no effective range *A*—it is set initially to the separation distance (*h*) for the last lag class graphed in the variogram. In the case of the spherical model, the effective range *A* = *A*_0_. In the case of the exponential model, the effective range *A* = 3*A*_0_, which is the distance at which the sill (*C* + *C*_0_) is within 5% of the asymptote. In the case of the Gaussian model, the effective range *A* = 3^0.5^*A*_0_, which is the distance at which the sill (*C* + *C*_0_) is within 5% of the asymptote.

The fractal dimension *D* was determined based on the log–log semivariogram plots using the formula^46^:

$$D = 2 - \frac{H}{2},$$

where: *H* is the slope of the semivariogram line plotted in the logarithmic system of coordinates.

Kriging.

The estimation of values in unmeasured places was conducted using the kriging estimation method. This method gives the best unbiased estimate of the point or block values of the variable under study with minimal variance during the estimation process. The values of the kriging variance depend on the position of the samples in relation to the estimated location, the weights assigned to the samples, and the parameters of the semivariogram model and is described by a linear equation expressed by the formula^45^:

$$z^{ \cdot } \left( {x_{o} } \right) = \sum\limits_{i = 1}^{N} {\lambda_{i} } z\left( {x_{i} } \right)$$

where *N* is the number of measurements, *z(x*_*i*_*)* is the measured value at the point *x*_*i*_, z*(x_*o*_*)* is the value estimated at the estimation point *x*_*o*_, and *λ*_*i*_ are the weights. The weights are determined from the system of equations taking into account the condition of non-loadability and efficiency of the estimator, i.e. when the expected value of the difference between the measured and estimated values is zero and the variance of the differences is minimal^45^:

$$\left\{ {\begin{array}{*{20}l} {\mathop \sum \limits_{j = 1}^{N} \lambda_{j} \gamma \left( {x_{i} ,x_{j} } \right) + \mu = \gamma \left( {x_{i} ,x_{o} } \right) i = 1 \ldots N} \\ {\mathop \sum \limits_{i = 1}^{N} \lambda_{i} = 1 } \\ \end{array} } \right.$$

Solving the above system of equations, we determined the weights of kriging—*λ*_*i*_. These weights allow also determination of the estimated value z* and its variance from the formula:

$$\sigma_{k}^{2} \left( {x_{o} } \right) = \mu + \sum\limits_{i = 1}^{N} {\lambda \,_{i} \gamma \left( {x_{i} ,x_{o} } \right)} .$$

Ordinary kriging (OK) was used for the estimation, as it gave a good match between the measured and the estimated value. The inverse distance weighting interpolation (IDW) was a worse interpolator, while the ordinary cokriging (OCK) did not appreciably improve the estimation compared to OK. Therefore, kriging maps were created using Gamma Design Software GS + 10^45^.

## Results

### Classical statistics

The statistical parameters of the examined soil characteristics in the studied region are summarised in Table 1. The mean saturated hydraulic conductivity of the soils was 2.597 m day^−1^ and ranged from 0.01 up to 11.54 m day^−1^. The average sand, silt, and clay contents and their ranges (in %) were 74, 24.5, 1.5 and 45–95, 4–54, 0–6, respectively. The silt content was always lower than that of sand and higher than that of clay at all sampling points. The content of organic carbon in the studied soils was low, i.e. on average 0.86% with the minimum and maximum values 0.002 and 3.75% at single measurement points. The reaction of the soils in general was either acidic or neutral with the mean, minimum, and maximum pH (in H_2_O) values of 5.3, 4.0, and 7.2, respectively. The mean cation exchange capacity (CEC) was 9.67 cmol kg^−1^ and ranged from 3.05 to 21.2 cmol kg^−1^. The average soil moisture measured with the TDR meter and gravimetrically was similar and amounted to approx. 0.07 (m^3^ m^−3^), whereas the respective ranges were 0.001–0.232 m^3^ m^−3^ and 0.009–0.287 m^3^ m^−3^. The mean, minimum, and maximum values of bulk density and total porosity were 1.414, 0.998,1.681 Mg m^−3^ and 0.424, 0.308, 0.524 m^3^ m^−3^, respectively. As in the study conducted by Dahiya et al.^44^ (1984), the variability was low for soil bulk density, total porosity, sand content, and pH in H_2_O (CV 8.8–13.8%), medium for CEC, silt content, OC, WC_TDR_, and WC_grav_ (32.1–67.4%), and high for saturated hydraulic conductivity (77.3%). Skewness, which characterises the degree of asymmetry of the distribution around the mean, was moderate (< 1) for most variables and slightly more positive (< 2) for soil moisture, clay content, and saturated hydraulic conductivity. Silt content and bulk density showed a slight negative asymmetry of (< – 1). Kurtosis, which characterises the relative slenderness or flatness of the distribution compared to the normal distribution (zero), was close to zero for most variables. We noted relatively little flattening for sand, silt, and pH (in H_2_O) (< 0 or from − 0.130 to − 0.158), slight slenderness for bulk density and porosity (< 0.053 or from 0.030 to − 0.030), and somewhat higher value for CEC (< 1 or 0.956). Soil moisture, the OC and clay contents, and saturated hydraulic conductivity showed much greater slenderness of distribution (2.218–4.826). The differences between the mean values and the medians for individual variables as well as the values of asymmetry and kurtosis indicate that the studied variables can be described with a normal distribution with fairly good accuracy. Those with greater asymmetry were square-root or natural-logarithm transformed, thus their data distributions were close to the normal distribution (Table 1).

**Table 1:** Basic statistics for soil properties at a depth of 0–20 cm in the study region.

Properties	N	Mean	Minimum	Maximum	SD	CV(%)	Skewness	Kurtosis
K	216	2.597	0.010	11.54	2.009	77.3	1.951	4.826
sand	691	74.0	45.0	95.0	9.3	12.6	0.094	– 0.130
silt	691	24.5	4.0	54.0	9.2	37.5	– 0.074	– 0.142
clay	691	1.52	0.0	6.0	0.905	59.4	1.537	4.183
pH_H2O_	691	5.27	4.04	7.2	0.728	13.8	0.680	– 0.158
OC	691	0.862	0.002	3.8	0.427	49.5	0.904	3.684
CEC	691	9.675	3.050	21.2	3.109	32.1	0.654	0.956
WC_grav_	228	0.067	0.009	0.287	0.045	67.4	1.626	3.586
WC_TDR_	228	0.071	0.001	0.232	0.038	53.7	1.330	2.218
BD	228	1.413	0.998	1.681	0.125	8.9	– 0.540	0.030
FI	228	0.425	0.308	0.525	0.038	8.9	0.000	– 0.030
**Transformed data with a square root (sqr) and natural logarithm (ln)**								
sqr(K)	216	1.507	0.100	3.400	0.573	38.0	0.640	1.080
sqr(clay)	691	1.166	0.000	2.450	0.403	34.6	– 0.610	2.570
ln(pHH_2_O)	691	1.653	1.400	1.980	0.134	8.1	0.410	– 0.510
sqr(OC)	691	0.895	0.040	1.940	0.248	27.7	– 0.520	1.490
sqr(CEC)	691	3.070	1.750	4.600	0.499	16.3	0.100	0.300
ln(WC_grav_)	228	– 2.914	– 4.710	– 1.250	0.667	22.9	– 0.200	– 0.250
sqr(WC_TDR_)	228	0.258	0.032	0.482	0.068	26.5	0.450	0.680

N—number of samples, SD—standard deviation, CV—coefficient of variation, K—saturated hydraulic conductivity (m day^−1^), sand—(2–0.05 mm), silt—(0.05–0.002 mm), clay < 0.002 mm, pH_H2O_—acidity (−), OC—organic carbon (%), CEC—cation exchange capacity (cmol kg^−1^), WC_grav_—gravimetric water content (m^3^ m^−3^), WC_TDR_—water content in time-domain reflectometry (m^3^ m^−3^), BD—bulk density (Mg m^−3^), FI—porosity (m^3^ m^−3^).

### Correlation analysis

The linear correlation coefficients (r) between the considered soil properties are summarised in Table 2 (the values marked in bold are statistically significant at *p* < 0.05). The saturated water conductivity of the soil was significantly positively correlated with the porosity (0.300) and negatively with the soil density (– 0.283). Other significant correlation coefficients were found between sand and silt contents (– 0.996) and sand and clay (– 0.182). There was no significant correlation between the contents of silt and clay. Soil pH (in H_2_O) and OC were negatively correlated (*p* < 0.05) with the sand content (– 0.177, − 0.175, respectively) and positively with the silt content (0.178, 0.168, respectively). CEC was negatively and significantly correlated (*p* < 0.05) with the sand content (– 0.519) and positively correlated with silt, clay, pH, and OC (0.160–0.607).

**Table 2:** Correlation coefficients (r) between soil variables in the study region.

Properties	sand	silt	clay	pH_H2O_	OC	CEC	WC_grav_	WC_TDR_	BD	FI	K
**Marked correlation coefficients are significant at p < 0.05, N = 216**											
sand	1.000	– **0.996**	– **0.181**	– **0.177**	– **0.175**	– **0.519**	– 0.060	– 0.068	– 0.084	**0.145**	– 0.034
silt		1.000	0.092	**0.178**	**0.168**	**0.470**	0.062	0.071	0.083	– **0.142**	0.034
clay			1.000	0.006	0.107	**0.607**	– 0.016	– 0.020	0.031	– 0.056	0.003
pH_H2O_				1.000	0.019	**0.160**	0.029	0.070	– 0.007	– 0.068	– 0.060
OC					1.000	**0.521**	– 0.039	– 0.016	– 0.078	0.007	0.062
CEC						1.000	– 0.013	– 0.005	– 0.006	– 0.079	0.034
WC_grav_							1.000	**0.876**	0.116	0.028	– 0.004
WC_TDR_								1.000	**0.192**	– 0.059	– 0.026
BD									1.000	– **0.735**	– **0.283**
FI										1.000	**0.300**
K											1.000

Sand—(2–0.05), silt—(0.05–0.002), clay < 0.002 mm, pH_H2O_—acidity (−), OC—organic carbon (%), CEC—cation exchange capacity (cmol kg^−1^), WC_grav_—gravimetric water content (m^3^ m^−3^), WC_TDR_—water content in time-domain reflectometry (m^3^ m^−3^), BD—bulk density (Mg m^−3^), FI—porosity (m^3^ m^−3^), K—saturated hydraulic conductivity (m day^−1^).

The significant correlation between the gravimetric vs. TDR soil moistures (0.876) indicates suitability of the TDR measurement system, which is widely used as a benchmark for validation of satellite soil moisture products (e.g.^47^). Soil moisture did not significantly correlate with other soil properties. Soil porosity correlated negatively (*p* < 0.05) with the bulk density and sand and silt contents (– 0.737, − 0.142) and positively with the sand content (0.145).

### Geostatistical analysis

The fitted semivariogram models for K and cross-semivariograms for pairs of cross-correlated K and other soil properties are presented in Table 3. In general, there was a good agreement between the theoretical exponential models for all soil properties and the empirical semivariograms, as indicated by the high values of the determination coefficients (R^2^ from 0.592 to 0.923) and the sum of squared residuals (RSS) from < 10^−6^ to 81.4 depending on soil properties. This agreement for the cross-semivariograms was fairly good in six cases (R^2^ > 0.284), and poor in two (R^2^ ~ 0.02). The RSS values were small for most models (5.94 × 10^−3^–7.62 × 10^−6^). In the cross-semivariograms analysis, five soil properties had exponential dependency, four—Gaussian, and one—linear. The presence of nugget effects indicates that the variability of the examined features is smaller than the adopted minimum distance between the measurement points. The sill values of the semivariance are comparable with the values of variance obtained in the classical way (Tables 1, 3), which may indicate lack of clear trends in the data. The sill values of the semivariograms were a derivative of the content of individual textural fractions. The highest sill values were recorded for the sand and silt contents. However, they were lower for saturated hydraulic conductivity and substantially lower for the contents of clay, organic matter, moisture, and pH, cation exchange capacity, bulk density, and total porosity. The range of spatial dependence displayed by the semivariograms was the smallest for pH (0.012°), intermediate for OC, CEC, clay, sand, silt, BD, WC_grav_, and WC_TDR_ porosity (0.018–0.057°), and the largest for saturated hydraulic conductivity (0.081°). In the case of the cross-semivariograms for pairs of cross-correlated K with intrinsic and relatively stable properties (sand, silt, clay, OC, CEC, pH), the spatial ranges were much larger (from 0.095° to 0.81°) than with dynamic ones, such as gravimetric and TDR soil moistures, bulk density, and total porosity (0.003°–0.009°). According to the classification of Cambardella et al.^48^, the spatial dependences (nugget/sill) for all semivariograms were moderate (0.25–0.75) and those for cross-semivariograms were in general strong (< 0.25). The distribution of the most widely studied soil properties showed anisotropy with orientation mostly from west to east. Only the clay content and CEC showed anisotropy from north to south.

**Table 3:** Fitted semivariogram models (SV) for properties data used in the ordinary kriging interpolation method and cross-semivariogram models (CSV) between saturated hydraulic conductivity and other soil properties; 1° corresponds to approx. 100 km.

Properties	Model	*C*_0_	*C*_0_ + *C*	C_0_/(*C*_0_ + *C*)	*A* (°)	R^2^	RSS	*A*_*z*_(°)	D0
**Semivariogram models (SV)**									
K	Exp	2.223	4.774	0.466	0.081	0.666	2.83E + 00	75	1.908
sqr (K)	Exp	0.187	0.374	0.499	0.063	0.592	1.26E−02	75	1.945
sand	Exp	41.4	89.57	0.462	0.024	0.911	8.14E + 01	96	1.945
silt	Exp	41.6	87.39	0.476	0.024	0.903	8.03E + 01	96	1.948
sqr(clay)	Exp	0.082	0.1714	0.478	0.024	0.733	6.35E−04	0	1.961
ln(pH_H2O_)	Exp	0.00563	0.01796	0.313	0.012	0.699	2.39E−06	124	1.973
sqr(OC)	Exp	0.0311	0.0631	0.493	0.021	0.923	1.25E−05	83	1.960
sqr(CEC)	Exp	0.1246	0.2532	0.492	0.018	0.904	2.32E−04	0	1.968
ln(WC_grav_)	Exp	0.1727	0.4294	0.402	0.018	0.568	5.78E−03	44	1.958
sqr(WC_TDR_)	Exp	0.00244	0.00489	0.499	0.057	0.605	1.82E−06	74	1.930
BD	Exp	0.00857	0.01724	0.497	0.045	0.609	1.40E−05	117	1.948
FI	Exp	0.00080	0.00149	0.541	0.045	0.782	5.56E−06	105	1.943

Properties	Model	*C*_0_	*C*_0_ + *C*	C_0_/(*C*_0_ + *C*)	*A* (°)	R^2^			
**Cross-semivariogram models (CSV) of sqr(K) and properties**									
sand	Gau	– 0.165	– 0.837	0.197	0.156	0.478			
silt	Gau	0.170	0.762	0.223	0.149	0.419			
sqr(clay)	Gau	0.0014	0.0463	0.030	0.240	0.316			
ln(pH_H2O_)	Lin	– 0.00066	– 0.00066	1.000	0.095	0.284			
sqr(OC)	Exp	0.00004	0.03148	0.001	0.810	0.177			
sqr(CEC)	Gau	0.0014	0.0863	0.016	0.222	0.394			
ln(WC_grav_)	Exp	– 0.0007	– 0.0235	0.030	0.006	0.013			
sqr(WC_TDR_)	Exp	– 0.00104	– 0.00286	0.364	0.009	0.020			
BD	Exp	– 0.00001	– 0.02102	0.000	0.006	0.295			
FI	Exp	0.00025	0.00604	0.041	0.003	0.237			

Exp.—exponential model, Lin.—linear model, Gau.—Gaussian model, *C*_0_—nugget variance, C_0_ + *C*—sill, *A*—effective range (°) (1° = approx. 100 km), R^2^—coefficient of determination, RSS—root sum square, *A*_*z*_—anisotropy (°—slope angle), D0—fractal dimension.

The estimation of the spatial distribution of the studied properties using the fractal theory showed that all soil properties were characterised by high values of the fractal dimensions D > 1.9, indicating a random distribution in the spatial organisation.

### Kriging maps

**Figure 2:**
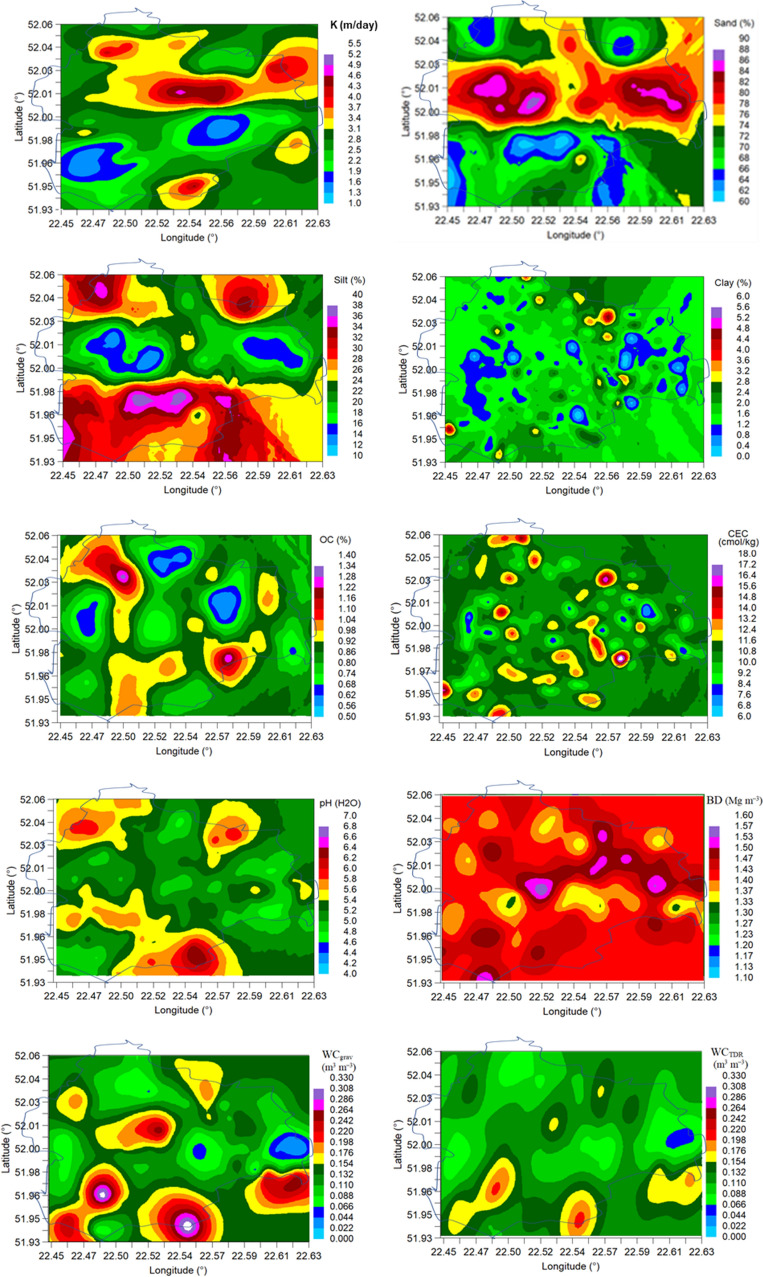
Maps of saturated hydraulic conductivity (K), sand, silt, and clay contents, soil organic carbon (OC), cation exchange capacity (CEC), pH, bulk density (BD), gravimetric water content (WC_grav_) and TDR water content (WC_TDR_). The study region is marked by a solid line.

Based on the obtained models, semivariogram parameters, and measured data, regional-scale maps of the soil properties were generated and the estimation errors were calculated (Fig. 2) using ordinary kriging. The estimation errors were 1–2% in the vicinity of the measurement points and up to approx. 10% at the edges of the estimated areas (measurement grids).

In the northern part of the study region, the large island (area) between approximately 52.01° and 52.04° with high saturated hydraulic conductivities from 3.1 to 4.6 m day^−1^ (Fig. 2) corresponds with the highest sand contents (> 74%) and the lowest contents of silt (< 22%). In the southern part of the region below 52°, the lower saturated conductivities correspond with the lower sand content (< 74%), greater silt content > 24%, and similar clay content (< 2.8%).

In general, latitudinal distribution can be observed for the sand and silt contents. Clay, CEC, and BD are distributed in a small island and OC, pH, and gravimetric and TDR soil moistures—in larger islands. Cation exchange capacity (CEC) showed a relatively uniform distribution throughout the study region in small islands of higher or lower values. Organic carbon content (OC) had mostly an island distribution system with a slightly marked meridional distribution. It can be observed that both gravimetric and TDR soil moistures were more similar to that of the organic carbon content than other soil properties. The gravimetric vs. TDR soil moisture distribution was more variable, which implies greater sensitivity. The highest BD values correspond with the highest sand content and the lowest contents of clay and OC.

## Discussion

### Geostatistical analysis

K exhibited the strongest spatial heterogeneity (coefficient of variation 77.3%) of all the soil studied properties. This can be largely influenced by various management practices, including crop rotation, tillage, liming, or history of land use and other treatments used at the regional scale. These management effects may be associated with changes in total porosity, which was significantly positively correlated with K (*p* < 0.05) (Table 2). Particularly important are large and connected (elongated) biological and inter-aggregate pores^9,16,17^. The spatially heterogeneous effect of the various management practices applied at the regional scale on K can mask the impact of intrinsic soil texture, as indicated by the poor and non-significant overall correlations between K and the contents of all textural fractions (Table 2). Probably, there was no significant effect of terrain attributes associated with the landscape level and pedogenetic processes on K, as the flat study area within the Łuków Plain is covered mainly by Podzol soils^49,50^.

The model parameters describing the spatial relationships of K with intrinsic and dynamic soil properties were better for the cross-semivariograms with auxiliary soil properties than for the direct semivariograms, as shown by the appreciably smaller nugget values (*C*_0_), the greater ranges (*A*), and the stronger degree of spatial dependencies (nugget/sill) in the former. These differences were most pronounced when intrinsic (stable) and relatively stable soil properties, including the contents of textural fractions, OC, and CEC, were used as auxiliary variables (*C*_0_ − 0.165 to 0.170 vs. 2.23), *A* 0.156° (15.6 km) to 0.810° (81 km) vs. 0.081° (8.1 km), nugget/sill 0.001 to 0.223 vs. 0.466). This indicates that the intrinsic and relatively stable soil properties were spatially correlated with K although there was no significant linear correlation of each intrinsic or relatively stable soil property vs. K (Table 2). The smaller nugget values (*C*_0_) in the cross-semivariograms compared to the direct semivariograms imply smoother spatial continuity and stronger dependency between neighbouring sampling points^31,51,52^. It is worth noting that the suitability of soil texture data used as auxiliary variables for improvement of the prediction of the spatial K distribution can be enhanced by their worldwide availability in soil geographic databases (e.g.^53^). It should be underlined that the range values of the cross-semivariograms (in the case of all pairs) exceeded the length and width of the study region (~ 13 × 16 km).

### Kriging maps

The kriging maps generated in this study allowed outlining two sub-areas with predominantly saturated hydraulic conductivity (K) > 3.0 m day^−1^ in the northern part (latitude 52.01–52.06) and < 3.0 m day^−1^ in the southern part (52.00–51.93) of the study region. As reported by Stryjewski^54^, the K values in the northern part can be classified as high and very high and those in the southern part as fairly high and low. The comparison of the maps in Fig. 2 shows positional similarity between the sub-area with the higher K value and those with the large sand content (> 74%) and the low silt content (< 22%). This similarity can be attributed to the effect of the sand fraction on the abundance of relatively large and connected pores that mostly contribute to high K (e.g.^55^). This effect can be illustrated by results from a study conducted by Lim et al.^56^, where K of 5.98 m day^−1^ of coarse sand decreased by 57, 88, and 96% with the successively decreasing sand content in fine sand, loam, and clay textured soils. Our previous studies in the same region along with visual observations showed that limited crop growth and yields were spatially related to higher sand content^50,57^. This crop response in sandier and more permeable zones can be caused by excessive drainage of rainwater resulting in insufficient plant-available water for unsaturated conditions. Furthermore, the drainage contributes to chemical leaching, thereby limiting the availability of nutrients for plants. This explanation can be supported by the significant negative correlation between the sand content and cation exchange capacity (Table 2). This implies that high K can be an indicator of a low-yielding zone in the studied area with predominance of coarse-textured soils. This is in contrast to fine-textured soils where low K values are indicative of low-yielding zones. For example, in a study conducted by Keller et al.^3^ on loam and clay soils with K varying from 0.6 to 25.2 m day^−1^, lower saturated hydraulic conductivity was recorded in low-yielding zones than in high- and medium-yielding zones due to the more blocky soil structure in the former. The low yields in fine-textured soils with low saturated hydraulic conductivity often results from water ponding and limited oxygen concentrations for root and shoot growth, especially in wet years^13^. This indicates that the effect of spatial distribution of K on the spatial distribution of soil productivity and other soil functions depends on soil texture and weather conditions during the growing season. Therefore, different threshold K values with respect to productivity and other soil functions should be considered in the case of coarse- and fine-textured soils.

The kriging map of K can be useful for the local authorities and agronomy advisers for spatial planning of management practices aimed at reduction of particularly high soil K. In coarse-textured soils, it can be reduced by addition of exogenous organic materials including biochar (as a soil conditioner)^27,56,58^ and locally available recycled composted chicken manure or spent mushroom substrate after mushroom harvesting^59,60^. Such organic materials are currently applied in the studied area in research and by some farmers on agriculturally used sandy soils.

Another important agricultural management practice influencing K is crop rotation, including green manure cover crops or intercropping systems^61,62^. This practice is subsidised in several countries, including Poland, to increase soil organic carbon content^63^, improve soil structure^64^, protect the soil surface from raindrop impact^65^, enhance fixation of atmospheric nitrogen in the case of legumes^66^, and improve agricultural productivity^62^. Over a longer time span, conversion of arable land into grassland that can serve as carbon and water storage may be an efficient option (e.g.^67^).

Also re-compaction of loose soil by traffic leads to reduced K due to a decrease and increase in the large and small pore volumes, respectively^68^. However, this practice needs to be applied with caution to avoid excessive soil compaction and its harmful effect on root growth and crop yield^13,69^. Saturated hydraulic conductivity values ≤ 0.1 m day^−1^ are used as an indicator of poor soil structure^70^ and more recently as threshold values of excessive soil compaction induced by vehicular traffic^71^. The K values in the study region were in general above the thresholds, which may be in part related to the presence of predominantly small farms where relatively light agricultural vehicles and implements are used.

## Summary and conclusions

The saturated hydraulic conductivity (K) of the soils in the studied region (140 km^2^) varied from 0.01 to 11.54 m day^−1^ and exhibited high spatial variability (CV 77.3%). This variability was higher than that of the contents of textural fractions and organic carbon, cation exchange capacity, soil water content, bulk density, and total porosity (CV 8.9–67.4%) (67.4% for grav. soil moisture and 53.7% for TDR soil moisture). K was significantly (*p* < 0.05) positively correlated with the total porosity (r = 0.300) and negatively correlated with the soil bulk density (r = – 0.283). The spatial prediction (autocorrelation) of the soil properties using single (direct) semivariograms varied from 0.012° (1.2 km) for pH to 0.081° (8.1 km) for K. Areas with larger K values are mainly determined by the proportion of sand and silt and those with smaller K—by the proportion of clay. The range of spatial dependence in the cross-semivariograms between K and sand and silt contents used as secondary variables was smaller (about 15 km) than that of cation exchange capacity, clay content, and organic carbon content used as secondary variables (about 22 to 81 km). However, the range of the spatial dependence prediction in the cross-semivariograms decreased when the dynamic soil properties (soil moisture, bulk density or total porosity) were used as secondary variables (0.3–0.9 km). The suitability of soil texture and organic carbon data to be used as secondary variables in cross-semivariograms for predicting spatial cross-correlations of K can be enhanced by their worldwide availability in soil geographic databases. The kriging maps allowed outlining two sub-areas with predominance of K > 3.0 m day^−1^ in the northern part (latitude 52.01–52.06) and < 3.0 m day^−1^ in the southern part of the study region. The comparison of the spatial maps indicates that there is positional similarity (agreement) between the sub-areas with the highest K values and the largest sand contents (> 74%). The spatial maps generated in this study can be helpful for the local authorities and agronomy advisers for spatial planning of management practices aimed at reduction of K of permeable and low-productive soils.
